# Assessment methods of pathological complete response in neoadjuvant head and neck cancer clinical trials: a scoping review

**DOI:** 10.1002/2056-4538.70114

**Published:** 2026-09-29

**Authors:** Peter Robinson, Charlotte Catrina Currie, Hisham Mehanna, Muhammad Shahid Iqbal, James O'Hara, Elsa Campôa, Myrto Moutafi, Max Robinson

**Affiliations:** ^1^ Faculty of Medical Sciences Newcastle University Newcastle upon Tyne UK; ^2^ Department of Cellular Pathology The Newcastle upon Tyne Hospitals NHS Foundation Trust Newcastle upon Tyne UK; ^3^ The Institute for Head and Neck Studies and Education (InHANSE) University of Birmingham Birmingham UK; ^4^ Northern Centre for Cancer Care The Newcastle upon Tyne Hospitals NHS Foundation Trust Newcastle upon Tyne UK; ^5^ Department of Otolaryngology ‐ Head and Neck Surgery The Newcastle upon Tyne Hospitals NHS Foundation Trust Newcastle upon Tyne UK; ^6^ Department of Oncology Unidade Local de Saúde do Algarve Portimão Portugal; ^7^ Department of Oncology Attikon University Hospital Athens Greece

**Keywords:** neoadjuvant, pathologic complete response, head and neck cancer

## Abstract

Head and neck cancer (HNC) trials have demonstrated pathological complete response (pCR) in patients receiving neoadjuvant treatment prior to surgery; however, systematic reviews have reported marked variation in pCR rates. This scoping review was designed to determine the definition, criteria, and assessment methodologies for pCR in interventional neoadjuvant HNC clinical trials. The review was conducted in accordance with the *JBI Manual for Evidence Synthesis* and the protocol prospectively registered. Medical databases, trial registries, and grey literature were systematically searched, and eligibility criteria applied. Study characteristics and pCR assessment methodologies were extracted and described. A total of 4,931 sources were identified, and screening identified 114 trials. Only 22 studies had publicly accessible protocols for assessment. Trials were initiated between 1989 and 2025, and the majority were phase II investigating chemotherapy + immunotherapy regimens (51%). pCR was most frequently classified as a secondary endpoint (55%), and reported pCR rates ranged from 0% to 100%. There was heterogeneity and incomplete documentation in both specimen preparation and pCR assessment. Where specified, most included assessment at both primary tumour and lymph nodes (50 of 69; 72%), and quantitative assessment calculating percentage residual viable tumour was common. While no formal guidelines for pCR assessment in HNC were identified, one trial outlined an immune‐related pathological response criteria for oral squamous cell carcinoma, and another included a detailed ‘pathological response assessment’ proforma. Citation of methodologies used in other cancers, for example, lung and breast cancer, was a recurring theme. Based on these findings, we propose pragmatic definitions, assessment considerations, and a structured reporting proforma to support increased transparency and reproducibility in pCR reporting. While these proposed elements are not consensus based, they provide a foundation for harmonisation efforts and highlight priorities for development of international consensus guidelines to enable meaningful comparison of interventions and determine the validity of pCR as a surrogate endpoint in HNC.

## Introduction

There is increasing interest in neoadjuvant regimens for head and neck cancer (HNC) as recent trials have demonstrated favourable outcomes [1, 2]. Results from the KEYNOTE‐689 phase III trial, published in 2025, demonstrated that the addition of neoadjuvant pembrolizumab and adjuvant pembrolizumab to standard of care treatment significantly improved event‐free survival, and both the UK National Institute for Health and Care Excellence and US National Comprehensive Cancer Network recently recommended this regimen as a standard of care option [2, 3, 4]. To date, the role of neoadjuvant treatment in HNC has varied worldwide with adoption by some institutions in select cases outside the clinical trial setting [5, 6, 7, 8, 9]. Although neoadjuvant regimens have been widely investigated internationally, the optimum regimen has not been defined and systematic reviews report variation in treatment modalities and clinical outcomes [10, 11]. As clinical trial data matures, neoadjuvant treatments will evolve and timely integration into standard of care requires reproducible validated endpoints to evaluate and compare efficacy.

Pathological complete response (pCR), broadly defined as absence of residual viable tumour (RVT) in a resected specimen following neoadjuvant therapy, has demonstrated prognostic value in several malignancies including breast, lung, and melanoma [12, 13, 14]. In HNC, pCR has been reported and also associated with favourable outcomes [15, 16, 17]. However, systematic reviews have reported variation in rates of pCR across HNC trials [10, 11]. To further stratify response following neoadjuvant treatment, alternative pathological response metrics including major pathological response (MPR; ≤10% RVT) and partial pathological response (pPR; ≤50% RVT) have been proposed. While these measures have demonstrated associations with disease‐free survival in some HNC studies, results remain inconsistent, and a clear association with overall survival has not been established [18].

In high‐risk early‐stage breast cancer, the validation and acceptance of pCR as a surrogate endpoint by the US Food and Drug Administration (FDA) was facilitated by harmonised definitions and assessment standards developed through the Collaborative Trials in Neoadjuvant Breast Cancer (CTneoBC) working group [19]. By contrast, the absence of formal consensus guidelines in other malignancies, including HNC, has been highlighted [20, 21, 22, 23]. Variability in specimen handling and assessment methodologies limit the utility of pCR as a surrogate marker of treatment effect and is a barrier to the adoption of novel treatment regimens into clinical guidelines.

While there are published systematic reviews comparing the outcomes of neoadjuvant interventions in HNC [10, 11], there are no reviews focussing on pCR methodology. A scoping review was designed to explore the assessment of pCR in this setting, with the aim of identifying key concepts, detailed protocols, and reporting guidelines [24].

## Methods

The review question was formulated using PCC framework:

- P (population): neoadjuvant HNC clinical trials
- C (concept): assessment methodology for pCR (including specimen requirements, specimen handling, reporting standards, reviewer criteria)
- C (context): worldwide interventional clinical trials investigating neoadjuvant therapy (all modalities) for the treatment of HNC, and reporting pCR as a trial outcome or reporting pCR in study results

The review question was: what is the definition, criteria, and methodology for assessing pCR in neoadjuvant clinical trials for HNC worldwide?

### Protocol and registration

The protocol was registered in the Open Science Framework repository [25]. The review has been reported in accordance with the *JBI Manual for Evidence Synthesis Scoping Reviews* and PRISMA‐ScR (Preferred Reporting Items for Systematic reviews and Meta‐Analyses: extension for Scoping Reviews) [26, 27].

### Eligibility criteria

Records were eligible for inclusion if they were interventional clinical trials investigating neoadjuvant treatment in HNC and either reporting pCR as a trial outcome or reporting pCR rate in the study results. Published guidelines which outlined assessment of pCR in HNC clinical trials were also eligible for inclusion. Records were excluded based on publication type (case reports, letters, systematic reviews, meta‐analyses), language (not written in English), and retracted records.

### Information sources and search strategy

Searches included electronic databases, clinical trial registries, grey literature containing databases, and targeted website searching. An initial search restricted to Embase® (OVID) and MEDLINE® (OVID) was conducted to develop a three‐domain search strategy; ‘neoadjuvant’, ‘pathological complete response’, and ‘head and neck cancer’ (Table 1). Terms within each domain were combined with ‘OR’ and the three domains combined with ‘AND’. The full search strategies for each database are available in the review protocol [25]. Search results were generated from database inception to 31 May 2025. All sources were searched on 5 June 2025.

**Table 1:** Search terms

Neoadjuvant (1)	neoadjuvant OR neo‐adjuvant OR induction chemotherapy OR ((presurgical OR preoperative OR perioperative) AND (chemotherapy OR immunotherapy OR treatment)) MeSH subject heading: Neoadjuvant Therapy/, Induction Chemotherapy/ Emtree subject heading: exp Neoadjuvant Therapy/, Induction Chemotherapy/
Pathological complete response (2)	patholog* complete response OR complete patholog* response OR patholog* complete remission OR complete patholog* remission OR pCR* MeSH subject heading: Pathologic Complete Response/ Emtree subject heading: Pathological Complete Response/
Head and neck cancer (3)	‘head and neck’ OR nasal OR sinonasal OR nasopharyn* OR hypopharyn* OR laryn* OR salivary OR ‘oral cavity’ OR tongue OR pharyn* OR parapharyn* OR oropharyn* OR mucosal MeSH subject heading: exp ‘Head and Neck Neoplasms’/ Emtree subject heading: exp ‘Head and Neck Cancer’/

### Selection process

Records from the database searches were imported into EndNote™ (Clarivate, Philadelphia, PA, USA) and duplicate records removed. Two reviewers (PR and CCC) independently screened the title and abstract of each record to determine eligibility. Inter‐rater agreement between the two reviewers was measured with Cohen's kappa (*κ*), and the strength of agreement was interpreted according to Landis and Koch [28]. The reviewers compared results and disagreements were resolved by consensus. Where a consensus could not be reached, disagreements were arbitrated by a third reviewer (MR). Articles that satisfied the inclusion criteria were subject to full‐text analysis. Articles not meeting the eligibility criteria after full‐text analysis were excluded, with reasons provided.

### Data charting

Two domains of data were recorded: study characteristics and pCR. Trial protocols, if publicly available, were retrieved for analysis. The specific data items for each domain (Table 2) were extracted independently in duplicate using pre‐piloted spreadsheets (Excel®; Microsoft, Redmond, WA, USA). This was followed by checks for concordance with resolution by consensus or with a third reviewer.

**Table 2:** Data extracted

Study characteristics	Trial identification number, study start date, origin country, sponsor, trial design, recruitment status, recruitment size, cancer subtype, neoadjuvant treatment modality, pCR rate
Pathological complete response	Endpoint classification, timing of surgery in relation to neoadjuvant treatment, assessment site(s), specimen handling/preparation, assessors, assessment methodology

### Data analysis and presentation

Data analysis was descriptive and focused to identify key concepts and methodologies in the assessment of pCR and highlight trends between clinical trials. Data were tabulated to facilitate synthesis of the information.

## Results

### Record selection

Database and clinical trial registry searching identified 3,394 records and grey literature searching identified 1,537 records; total 4,931. After the removal of duplicate records, the titles and abstracts of 3,777 records were screened independently by two reviewers; 3,502 were excluded (inter‐rater agreement Cohen's kappa statistic *κ* = 0.76, substantial agreement). The remaining 275 records were subject to full‐text analysis; 58 were excluded (inter‐rater agreement Cohen's kappa statistic *κ* = 0.61, substantial agreement), leaving 217 eligible records representing 114 individual trials. The PRISMA flowchart details the screening and selection process with reasons for exclusion (Figure 1). Forward and backward citation searching were omitted as there were 114 unique clinical trials to assess.

**Figure 1:**
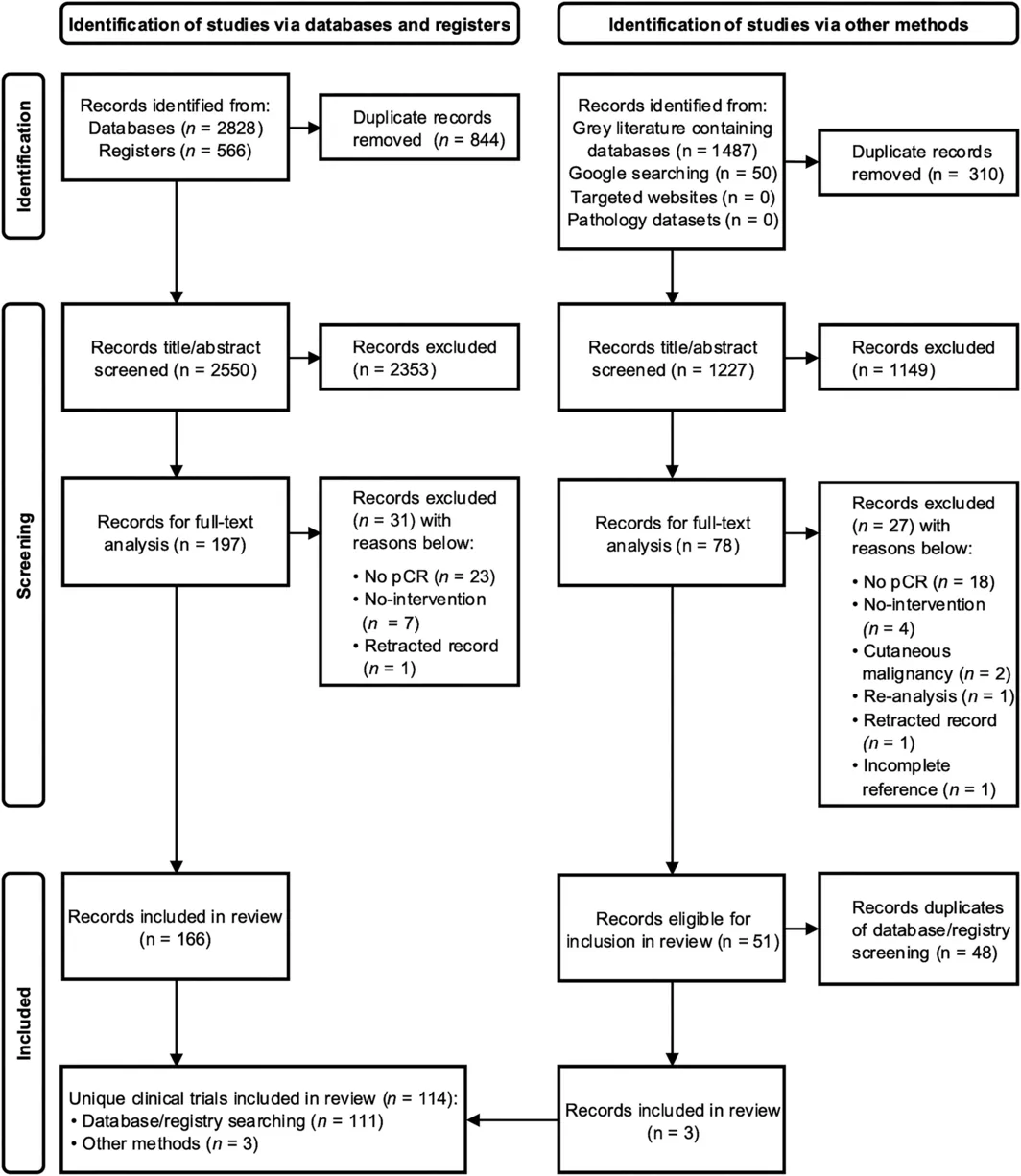
Record screening. pCR: pathological complete response. Databases: MEDLINE® (OVID) 1,272; Embase® (OVID) 760; Cochrane Library 266; Scopus 528; Web of Science 2. Registries: clinicaltrials.gov 537; WHO International Clinical Trials Registry Platform (ICTRP) 9; ISRCTN 6; EU Clinical Trials Register 14. Grey literature containing databases: WHO Institutional Repository for Information Sharing (IRIS) 149; Bielefeld Academic Search Engine (BASE) 375; TripPro 963.

### Clinical trial characteristics

Of the 114 clinical trials that met the eligibility criteria, only 22 studies had publicly accessible protocols for assessment. Trials were initiated over the period 1989 to 2025 with an increase in trials referencing pCR from 2021. At the time of data extraction, 64% of studies were still active and 31% were completed. Most studies were initiated in either China (62%) or the United States (20%). The majority of were phase II (80%), single‐arm design (60%), and investigating locally advanced HNC. The most frequently investigated neoadjuvant regimen was chemotherapy combined with immunotherapy (60%). Other systemic regimens included chemotherapy only (12%) and immunotherapy only (19%). Radiotherapy was included in the neoadjuvant protocol in 15 trials (13%). One study evaluated two radiotherapy interventions, resulting in 16 radiotherapy‐containing regimens overall. Of these, two investigated radiotherapy alone, two chemoradiotherapy, eight immunoradiotherapy, and four combined radiotherapy, chemotherapy, and immunotherapy. The individual neoadjuvant regimens for each trial are provided in the supplementary material, Table S1. Seventeen trials included a comparator arm with no neoadjuvant treatment (i.e. surgery ± adjuvant treatment). pCR was classified as a secondary endpoint in around a half of trials (55%) and 12 trials (11%) did not specify pCR as a trial endpoint but reported pCR in results. One study outlined the rationale for pCR as a secondary endpoint citing that ‘pCR can be rare in other cancers, which may restrict its use as a predictive endpoint’. [29] One trial changed their primary endpoint from pCR to MPR without a documented rationale [30]. Reported pCR ranged from 0% to 100%. The characteristics of the 114 included trials are provided in the supplementary material, Table S1.

### Assessment of pCR

No formal guidelines for pCR assessment in HNC were identified. Trials demonstrated incomplete documentation of pCR assessment methodologies. When documented, variation in the definition and assessment of pCR was observed. Timing of surgery or biopsy ranged from the day after to 13 weeks after completion of neoadjuvant treatment but was not clearly described in around half of trials (46%) [30, 31].

#### Definition of pCR

Almost half of trials did not provide a pCR definition (49%). When reported there were inconsistencies in the wording used to define pCR. Definitions frequently employed composite or atypical phrasing combining qualitative descriptors, quantitative metrics, and anatomical criteria (e.g. ‘no invasive and no in situ residuals’, ‘0% residual tumour’, ‘assessment of the primary site with or without lymph nodes’; supplementary material, Table S1). One trial defined pCR as ypT0N0 using the Union for International Cancer Control (UICC) TNM 8th Edition classification [32].

#### Assessment sites

Precise documentation of the sites assessed for pCR was not described in 39% of trials. Where documented, the majority of assessments included both the primary site and lymph nodes (50 of 69; 72%). Assessment of the primary site only was documented in 12 trials, and seven had unclear documentation referring only to ‘resection specimen’ without further details. Interestingly, one trial outlined assessment at ‘primary site’ but the evaluation included ‘resected specimen and all sampled ipsilateral lymph nodes’. [33] Another trial outlined a rationale for excluding assessment of lymph nodes: ‘given that pre‐treatment biopsies of lymph nodes were not performed and thus would be unavailable for comparison’ [34]. Some trials also described assessment of pCR on biopsies as opposed to resection specimens [35, 36, 37]. There were changes between planned assessment sites and reported results; in one trial pCR rate was only reported for lymph nodes and did not include the primary site, which was described in the trial registration [38, 39].

#### Specimen handling and preparation

The descriptions of specimen handling and preparation were varied, with 80 trials not outlining any methods (supplementary material, Table S1). Trial protocols referenced external documents containing further details on processing, such as Pathology Manuals, Laboratory Manuals, and Vendor Manuals, although none were publicly available for assessment [29, 40, 41]. Specimen handling procedures were documented in three published journal articles but not included in the trial protocols [41, 42, 43]. For specimen dissection, one block/cm was most frequently specified, however smaller increments of 5 and 3 mm were also described [43, 44, 45, 46]. Differences in preparation of primary sites with and without discrete mass lesions was outlined in one trial; discrete lesions would have the ‘entire mass submitted for evaluation’, whereas a primary without a discrete lesion would have the ‘entire specimen examined’. [47] Preparation methods used in other malignancies, such as lung cancer, were cited [46, 48]. Re‐examination of the ‘entire tumour bed’ when no residual tumour could be identified under microscopic evaluation was specified in NeoRTPC02 [43]. Trials with central pathology review had variability in the type and extent of tissue acceptable for assessment. One trial outlined preference for submission of the entire primary tumour and lymph nodes [49]. Trials differed in slide review requirement with some requesting all slides prepared from blocks, whereas others required only representative slides meeting predefined criteria [29, 30, 50]. Evaluation on haematoxylin and eosin staining was outlined in 12 trials and two trials included immunohistochemistry (e.g. CD4, CD8, CD20) for pCR assessment [43, 51].

#### Assessors

Nine trials specified central pathology review and 11 included multiple independent assessors. Blinding of pathologists to participant data was outlined explicitly in nine trials (supplementary material, Table S1). In one trial, if the calculated percentage of tumour differed by ≥10% between two assessors, a review by a third pathologist was required [43]. Requirement of at least 10 years' experience was specified in two trials and completion of formal training outlined in one trial [29, 48, 52].

#### Assessment methods

Three trial protocols outlined detailed requirements of pathology data to be collected, including staging, pathological response, percentage of viable tumour cells, number of lymph nodes removed at each level, evaluation of extra‐capsular nodal spread, perineural and vascular invasion [30, 40, 50]. One trial included a comprehensive ‘Post‐surgery pathological response assessment’ proforma within the ‘Pathology assessment guidelines’. [40] The proforma included structured documentation of perineural invasion, lymphovascular invasion (LVI), number of lymph nodes, and incorporated percentage‐based quantification of viable tumour, necrosis, stroma, and foreign body reaction to calculate pathological response.

#### Quantitative assessment

Quantitative assessment of pCR was described in several trials, most commonly using percentage residual viable tumour (%RVT). The immune‐related pathological response criteria (irPRC) developed for lung cancer were the most cited methodology [53]. Other trials referenced methodologies from previous HNC trials or adapted approaches used for lung cancer, breast cancer, and melanoma [15, 22, 23, 36, 54, 55].

%RVT was calculated using [RVT surface area/total tumour bed × 100] with the tumour bed commonly defined as the sum of RVT, tumour‐associated stroma, necrosis, and regression bed. pCR was defined as 0% RVT [23]. An alternative method involved calculation of pathological treatment effect (PTE), calculated by [areas of treatment response/total tumour surface area × 100] with pCR corresponding to 100% PTE. One trial described a pathology‐enhanced Response Evaluation Criteria in Solid Tumors (RECIST) methodology that incorporated both histological and imaging‐based assessment [56].

There were discrepancies in rounding of quantitative analysis percentages. While one trial specified rounding to the nearest 1%, other trials reported in 10% increments, and one trial required 10% increments except for values below 10% which were recorded as single digits percentage between 1% and 5% [40, 48, 57]. Utilisation of digital pathology was described in four trials to enable slide annotation to distinguish viable tumour from areas of treatment effect to automatically quantify %RVT or PTE [49, 57].

#### Qualitative features of pCR

Several trials outlined histological features that would classify as pathological response (Table 3). Commonly described features included necrosis, fibrosis, giant cell reaction, histiocytic inflammation, and immune infiltration. Some trials referenced features described in previous HNC studies, such as Licitra *et al* [15] and Uppaluri *et al* [58], others referenced features taken from rectal and breast cancer [59, 60]. One trial proposed an irPRC for oral squamous cell carcinoma adapted from irPRC described for lung cancer [46]. The features included: ‘multinucleated giant cell infiltration, dystrophic calcification, tumour infiltrating lymphocytes (TILs), foamy macrophages, neovascularisation, proliferative fibrosis, tertiary lymphoid structure, and dense plasma cells’. [46] Scanned slides annotated with areas of RVT, pathological response features, and tumour bed were included in the supplementary material for two trials [46, 61].

**Table 3:** Histological features included in descriptions of pathological response

Non‐viable tumour
Necrosis
Fibrosis
Neovascularisation
Granulation tissue
Tumour infiltrating lymphocytes
Lymphocyte infiltration
Histiocytic inflammation
Foamy macrophage reaction
Cholesterol clefts
Giant cell reaction
Multinucleated giant cell infiltration

## Discussion

Rather than evaluating the clinical efficacy of neoadjuvant regimens, we have focused on how pathological response is defined, assessed, and reported in neoadjuvant HNC clinical trials. There were inconsistencies in the definition of pCR, with different terms being used, details such as timing of surgery were frequently omitted, anatomical site evaluation was inconsistent, and there was heterogeneity in specimen handling protocols and reporting of histological features.

Early clinical trials (1989–2008) identified by our review predominantly evaluated chemotherapy‐based regimens. Several randomised controlled trials from this period did not demonstrate an overall survival benefit compared with upfront surgery or reported a high incidence of treatment‐related toxicity which may have limited adoption [15, 17, 62]. The advent of immunotherapy, alone or with chemotherapy, together with increased understanding of disease mechanisms has transformed the therapeutic landscape in HNC [63]. The positive results from KEYNOTE‐689 and subsequent incorporation into treatment recommendations increase the need for consensus guidelines to support standardised and reproducible pathological response assessment [2].

Where methods were described, assessment of both primary site and lymph nodes was most common. However, there was inconsistency in specified sites; in one trial ‘primary site’ included lymph nodes, whereas in other trials lymph nodes were excluded, or there was ambiguity [33]. For trials enrolling both ‘node‐positive’ and ‘node‐negative’ participants, failure to clearly define nodal assessment criteria may significantly affect interpretation of pCR rates.

Although pCR was most commonly defined as 0% RVT, differences in inclusion of lymph nodes, histological features of response, and mathematical rounding could influence reported rates. Definitions that exclude nodal assessment or permit residual *in situ* disease may inflate pCR rates and weaken associations with long‐term outcomes. Defining pCR as ypT0N0, a staging‐based approach, may underestimate meaningful biological response, especially in immunotherapy‐based regimens where %RVT can capture the treatment effect and not just tumour presence [64]. Methodological heterogeneity may contribute to the wide range in pCR rates reported and makes comparison of interventions through meta‐analyses problematic.

Quantitative assessment using %RVT and PTE was often adapted from other malignancies, most frequently irPRC for lung cancer [53]. However, differences in tumour biology, immune microenvironment, and response to therapy between cancer types may limit generalisability to HNC. Only one trial proposed an oral squamous cell carcinoma‐specific irPRC, detailing a disease‐specific histological response pattern [46]. Comprehensive documentation of features associated with pathologic response may facilitate identification of independent prognostic factors in HNC, similar to those discovered in neoadjuvant response patterns in breast cancer [65].

Although some trials explicitly specified documentation of LVI, which is a core item in HNC reporting datasets, its influence on pathological response categorisation remains uncertain [30, 40, 50, 66, 67]. Similarly, the role of TILs, which have been observed in treatment responders and may have predictive significance, has not been defined [50, 67, 68, 69]. Other individual features, specifically residual *in situ* disease, isolated tumour cells in lympho‐vascular spaces alone, and isolated tumour cells and micro‐metastases in lymph nodes, lack the required granularity of reporting to establish their significance in pathological response assessment. In the absence of high‐level evidence for inclusion of these parameters in pathological response assessment, further guideline development by expert consensus opinion is required to facilitate methodological consistency and standardised pathological response reporting.

As demonstrated in breast cancer, a standardised and transparent assessment methodology to facilitate interpretation and reporting has enabled regulatory recognition of pCR as a surrogate endpoint [19]. The International Association for the Study of Lung Cancer (IASLC) consensus guidelines integrate quantitative RVT assessment capturing biological treatment effect [21]. By comparison, in HNC, absence of consensus definitions and inconsistent documentation across registries, protocols, and publications represent barriers to clinical guideline adoption (Table 4). Discrepancies in documentation between trial registries, protocols, and publications were observed. Transparent reporting and public availability of methodologies would improve reproducibility of pCR assessments and aid trial design where pCR is used to guide interventions [70, 71].

**Table 4:** Comparison of pathological response frameworks

	Breast cancer (CTneoBC) [19]	Lung cancer (IASLC) [21]	Head and neck cancer
Framework status	Internationally standardised	International consensus	No consensus framework
pCR definition	No residual invasive cancer ± *in situ* disease in primary ± nodes (ypT0/Tis ypN0 or ypT0 ypN0)	0% RVT in tumour bed	Variable (ypT0N0, 0% RVT, qualitative terms)
Specimen handling	Harmonised guidelines	Detailed, standardised protocols	Variable and often not documented
Quantitative assessment	Binary (pCR or no pCR)	%RVT	Inconsistently applied
Predictive value	Yes	Yes	Not established
Surrogate endpoint status	Regulatory acceptance (FDA)	Not validated	Not validated

It is unclear if the introduction of MPR as opposed to pCR as a measure of efficacy reflects the possibility that pCR is an uncommonly observed phenomenon in certain HNC subtypes [2, 30]. Determining if pCR and/or MPR correlate to long‐term outcomes will require harmonised assessment methods. Future research should also evaluate radiologic‐pathologic response correlation and the potential role of digital pathology and artificial intelligence in standardising and quantifying pathological response.

Based on our scoping review, we suggest definitions, specimen handling, assessment, reporting, and quality assurance methods (Table 5) and a structured pathological response reporting proforma (Table 6). The proforma is intended as a starting point to facilitate calculation and reporting of pathological response and is recommended to be used alongside validated histopathology datasets to support transparent documentation and reporting in clinical trials. The significance of some individual features in the assessment of pathological response and subsequent assignment to response categories requires formal guideline development by expert consensus opinion and will inform further iterations of the proforma. As neoadjuvant regimens become standard of care, routine documentation of pathological response will become clinically relevant; however, the assessment methodologies and prognostic significance of the response categories remain to be defined outside the clinical trial setting [18].

**Table 5:** Suggested definitions and assessment methods

**Definitions** Pathological complete response [pCR; 0% Residual Viable Tumour (RVT)]: the absence of residual invasive cancer on haematoxylin and eosin evaluation of the primary resection specimen and all sampled regional lymph nodes following completion of neoadjuvant systemic therapy (i.e. ypT0 ypN0 in the current AJCC/UICC staging system) [19]. Major pathological response (MPR; ≤10% RVT): no more than 10% residual viable invasive cancer within the resected primary tumour specimen and all sampled regional lymph nodes [2, 18]. Partial pathological response (PPR; ≤50% RVT): no more than 50% residual viable invasive cancer within the resected primary tumour specimen and all sampled regional lymph nodes [18].
**Specimen handling** Assessment at primary site and neck dissection(s) if performed. For cases with a discrete lesion at primary site, the entire lesion is submitted for histological evaluation. If no residual tumour is identified during microscopy, the entire specimen should be submitted for histological assessment. For case without a discrete lesion at primary site, the entire specimen is submitted for histological evaluation [47]. One block per 1 cm of the discrete lesion or submit the entire specimen. Recommended block thickness ≤3 mm and one section per block. Haematoxylin and eosin staining, immunohistochemistry not currently recommended.
**Assessment** Pathologic response assessed quantitatively using %RVT (%RVT = RVT surface area/total tumour bed surface area × 100). The tumour bed defined as RVT + tumour stroma + regression bed. Qualitative features of pathological complete response should be documented. The features of the regression bed are outlined in Table 3. In the future, validated digital pathology and image analysis methods may improve accuracy and reproducibility.
**Reporting** %RVT at primary site and lymph nodes reported separately. Overall %RVT is the average of the primary site %RVT and lymph nodes %RVT. If there are no lymph node metastases record %RVT at primary tumour. Use a structured reporting proforma (Table 6).
**Quality assurance** For clinical trials, central review is recommended. Entire slide set from participating sites to be reviewed by lead pathologist or delegated pathologist(s).

**Table 6:** Structured pathological response proforma* ^,^ ^†^ ^,^ ^‡^

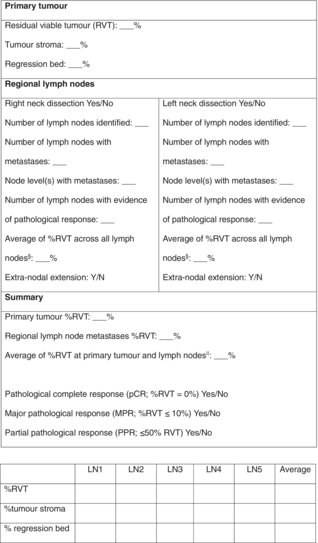

* Features of pathological response are listed in Table 3. Histological examples of the tumour bed and regression bed are provided by Stein *et al* [23].

^†^ %RVT + %tumour stroma + %regression bed should equal 100%.

^‡^ %RVT recorded in 10% increments, except values <10% where single figures recorded.

^§^Assess each lymph node separately and then calculate an average.

^||^If no lymph node metastases, record %RVT at primary site.

### Limitations

This scoping review has been transparently reported in accordance with the *JBI Manual for Evidence Synthesis Scoping Reviews* and PRISMA‐ScR [26, 27]. Deviations from the protocol include removal of citation searching (Step 3) owing to the identification of 114 through Step 2 searching. Selection bias of sources was minimised by searching medical databases, trial registries, and grey literature, and utilisation of two reviewers who independently screened and selected studies for inclusion. The eligibility criteria, which excluded articles not written in English, could have led to selection bias during identification of trials. Incomplete reporting and lack of details provided for pCR methodologies could have hampered identification assessment methods. Principle investigators were not contacted for protocols or additional manuals. As this is a scoping review, a quality assessment of trials was not performed.

## Conclusions

This scoping review identifies inconsistent and incomplete documentation of pCR methodologies in interventional neoadjuvant HNC clinical trials. To address these gaps, we summarise recurring methodological elements from the existing literature and propose pragmatic definitions, assessment considerations, and a structured reporting proforma to support more transparent and reproducible pCR reporting in future trials. While these proposed elements are not consensus based, they provide a foundation for harmonisation efforts and highlight priorities for the development of international, multiprofessional consensus guidelines, which will be essential to enable robust comparison of interventions and to determine the validity of pCR as a surrogate endpoint in HNC.

## Author contributions statement

MR and PR conceived the study. All the authors contributed to the development of the protocol. PR and CCC extracted, analysed and synthesised the data. MR and PR wrote the first draft of the manuscript, and all authors critically reviewed and approved the final version of the manuscript.

## Supporting information


**Table S1.** Trial characteristics and pathological complete response assessment methods
